# COVID-19 Therapy: Could a Copper Derivative of Chlorophyll *a* Be Used to Treat Lymphopenia Associated With Severe Symptoms of SARS-CoV-2 Infection?

**DOI:** 10.3389/fmed.2021.620175

**Published:** 2021-03-12

**Authors:** Nicole F. Clark, Andrew W. Taylor-Robinson

**Affiliations:** ^1^Institute for Applied Ecology, University of Canberra, Bruce, ACT, Australia; ^2^Centre for Marine Bioproducts Development, College of Medicine and Public Health, Flinders University, Bedford Park, SA, Australia; ^3^Infectious Diseases Research Group, School of Health, Medical and Applied Sciences, Central Queensland University, Brisbane, QLD, Australia; ^4^College of Health and Human Sciences, Charles Darwin University, Casuarina, NT, Australia

**Keywords:** chlorophyll, therapy, lymphopenia, SARS-CoV-2, COVID-19, coronavirus, sodium copper chlorophyllin

## Introduction

Chlorophyll *a* is a specific form of chlorophyll involved in oxygenic photosynthesis. It contains a magnesium ion surrounded by a large ring structure known as a chlorin. Four nitrogen atoms from the chlorin encase and bind the magnesium atom. The magnesium center uniquely defines the molecule as chlorophyll *a* (1). In order to harness the widely acknowledged therapeutic benefits of chlorophyll *a*, a chemical process known as re-greening must occur whereby the central atom is replaced with another metal yielding the same electrostatic charge, such as zinc or copper (2). Dietary chlorophyll, a formulation derived from sodium copper chlorophyllin (SCC), is a popular dietary supplement taken by health-conscious consumers (3, 4). Chlorophyll *a* derivatives including SCC are known to have a number of benefits when taken at therapeutic doses (3, 5). They are non-toxic, highly soluble compounds that are demonstrated to have higher uptake in human cell systems which likely triggers the chelation of ionic compounds (6, 7).

Several chlorophyll *a* derivatives have a profoundly cytotoxic effect *in vitro* and *in vivo* when compared to controls (5). Due to the antioxidant potential of chorophyllins double-blind placebo-controlled trials have revealed significant therapeutic outcomes, most notably in the prevention and treatment of cancers (5, 8–11). However, the therapeutic efficacy in treating numerous conditions within a broad range of clinical settings, particularly in countries like the US, UK, Canada and Australia, is largely neglected. For the recently emergent Corona Virus Disease-2019 (COVID-19) pandemic that is caused by infection with the novel human pathogen Severe Acute Respiratory Syndrome (SARS) Coronavirus (CoV)-2 (12) it was proposed that zinc chlorophyll may show potential as a therapeutic (13). This is because this tetrapyrrole derivative may aid the uptake and free ionization of zinc and thus potentially inhibit ribonucleic acid synthesis of SARS-CoV-2 in human epithelial lung tissue. Much like zinc chlorophyll, SCC is a non-toxic, water-soluble chlorophyll *a* derivative that may offer therapeutic benefits against SARS-CoV-2 but for entirely different reasons.

The therapeutic efficacy of SCC in both animals and humans via oral and parenteral routes, particularly intravenous infusions, is well-documented (3). Furthermore, its capacity to inhibit viral cytopathicity *in vitro* has been demonstrated (14). SSC exhibits significant anti-viral properties against a number of pathogenic viruses including the causative agents of highly infectious respiratory diseases such as influenza (14). A recent review suggested that in common with other copper-based compounds SCC could act as an anti-viral agent in the treatment of COVID-19 (15). Whilst the anti-viral property of zinc is established (16), vanishingly few studies consider the function that other metal ions may play in humoral immunity (17). In this context copper is an essential co-factor at the site of blood stem cell production and is thus instrumental in stimulating haematopoiesis (18–20).

As copper plays a key role in the production of leukocytes its deficiency has been linked to the condition of leukopenia, a reduced number of leukocytes in the peripheral blood, especially of neutrophils but also lymphocytes and granulocytes (21, 22). A limited number of reports have examined copper deficiency and the role it may play toward individual immunity and susceptibility to human diseases (23–25), while a few studies have investigated the importance of copper and copper-based compounds such as SCC in maintaining leukocyte homeostasis. Restoration of serum copper levels by intravenous infusion of patients led to a rise in peripheral blood leukocytes (18–20), highlighting the link between metabolic copper deficiency and susceptibility to disease. Similarly, likely due to the fact that it contains copper, SCC has also been shown to significantly increase leukocyte levels in a range of patients (10, 26–28), revealing the potential to treat those afflicted with leukopenia due to other diseases or disorders. Despite this cumulative clinical evidence, however, the therapeutic application of copper and chlorophyll *a* derivatives such as SCC, particularly with regard to acute leukopenia caused by viral infections, is rarely deliberated. Moreover, to date the use of SCC to treat aggressive lymphopenia [also called lymphocytopenia, an abnormally low concentration of lymphocytes in the peripheral blood, ≤ 1,100 cells/μL; (29)], a major feature of SARS-CoV-2 infection, has not been considered.

## SARS-CoV-2 and Lymphopenia

At the time of writing there are over 109,140,000 confirmed cases of COVID-19 globally, of which close to 2,408,000 have proved fatal (30). Maintaining a satisfactory peripheral blood lymphocyte count (between 1,100 and 4,800 cells/μL in adults) is a key contributory factor in the survival of COVID-19 patients, such that lymphopenia is noted as an associated risk factor in COVID-19-related deaths (31–33). Hence, in the current absence of a regulatory authority-approved drug for use against severe cases of COVID-19 lymphopenia is a surrogate indicator of a patient's poor prognosis (29). This outcome is thought to be as a consequence of the overexpression of interleukin (IL)-6 (34). This activates unregulated proliferation of leukocytes, particularly first responders such as neutrophils, followed only later by macrophages and lymphocytes (32, 35). The cellular expansion triggers a pro-inflammatory cytokine storm that leads to excessive inflammation, destruction of epithelial tissue and pulmonary oedema (36–38), eventually resulting in cardiac arrest due to depletion of oxygen concentrations in the blood (39–41).

Symptomatic COVID-19 patients show significant depletion of peripheral blood leukocytes, in particular presenting with lymphopenia when compared to asymptomatic or mild disease-presenting patients, suggesting greater disease severity correlates to a progressive reduction in lymphocytes (33, 41–43). In one corroborative study elevated neutrophil levels correlated to a significant reduction in the proportion of the total leukocyte population that comprised lymphocytes (31). This is likely due to a rapid innate immune response involving neutrophils (41, 43), since they are the most numerous and most abundant leukocyte early in infection. Also, when compared to lymphocytes, which take longer to mature and have a more specialized response (33), less metabolic expenditure is required to produce neutrophils. This may explain why as an apparent “last ditch” effort to survive some terminally ill COVID-19 patients overproduce neutrophils (31).

A symptom of many infectious diseases, leukopenia is contraindicated with life expectancy; thus, lower concentrations of peripheral blood leukocytes, in particular CD4^+^ and CD8^+^ T lymphocytes, are often surrogate indicators of disease severity (44). Perturbation of leukocyte homeostasis, specifically lymphopenia, predicts disease severity among symptomatic COVID-19 patients (38). In contrast, individuals who test positive for SARS-CoV-2 but are asymptomatic for COVID-19 do not present with low or reduced peripheral blood lymphocyte levels, which thus places them at a lower risk of life-threatening COVID-19-related complications (41). Immunocompromised persons are also at increased risk from COVID-19 due to their insufficient numbers of lymphocytes (42). This means that disease outcome is strongly associated with immunological response and thus increased risk is linked indirectly to perturbed haematopoiesis. An inadequate production of lymphocytes could be a direct result of the disease itself or due in part to pre-existing states such as old age and underlying immunocompromised conditions (31, 45). This suggests that maintaining adequate peripheral blood lymphocyte levels may control symptoms and disease severity of COVID-19 patients (40). This is achieved through preventing excessive production of neutrophils and overexpression of IL-6 (34), thereby averting the characteristic pro-inflammatory cytokine storm and potentially fatal pulmonary oedema that would otherwise ensue (40).

## Sodium Copper Chlorophyllin and COVID-19

Irrespective of whether it is pre-existing or triggered by exposure to SARS-CoV-2, peripheral blood lymphopenia predicts disease severity in COVID-19 patients (32). Finding ways to reduce as much as possible this critical immune-modulated deficit may improve treatment outcomes of symptomatic or at-risk individuals (40). In both humans and animals therapeutic doses of SCC significantly increase peripheral blood leukocyte levels (10, 46–48). Clinical trials in patients, including children suffering from leukopenia due to cancer-related illness (28), demonstrated that an oral dose of SCC taken at 180 mg for adults and 40 mg for children three times daily significantly increased whole leukocyte concentrations, particularly neutrophils, compared to controls (26–28). Another study indicated this to be as effective as the standard treatment leucogen used to control neutropenia (10). Increases in leukocyte counts of > 30% in 2 weeks and 82% after 1 month were reported. Restoration occurred for 85% of the subjects, with extremely minimal side effects and no noted toxicity. As participants in each of these trials were treated not for lymphopenia but instead neutropenia, unfortunately the concentration of lymphocytes was not recorded (10, 26–28). Overall, however, these findings suggest that similar therapy may be effective against SARS-CoV-2, as restorative activity of lymphocytes could occur before severe symptoms of COVID-19 appear.

Support for this proposal comes from murine models in which administration of SCC was demonstrated to significantly increase the peripheral blood concentration of lymphocytes (47, 49). It is therefore entirely possible that in human subjects a marked lymphocytosis induced by therapeutic doses of SCC, delivered orally or parenterally, may also be observed. Furthermore, SCC significantly suppresses IL-6, as shown by studies *in vitro* and *in vivo* (48, 50). For patients infected with SARS-CoV-2, SCC could be utilized to ameliorate aggressive immune-modulated outcomes by suppressing pro-inflammatory cytokine effects through blocking trans-signaling of IL-6, thereby leading to a reduction in lymphocytes and an overproduction of neutrophils (48, 50). Once a person is exposed to SARS-CoV-2 several days may elapse before they become symptomatic, if at all, and several more before severe symptoms develop (39). Hence, treatment with SCC at the time of onset of symptoms and/or at diagnosis, especially for immunocompromised patients, may control leukocyte levels contraindicated with disease severity. COVID-19 symptoms often start to worsen by days 10–12 after virus exposure while intensive care unit admission typically occurs from days 12–14 (36, 39). Therefore, taking SCC before the disease progresses to this point may prevent functional exhaustion of CD4^+^ and CD8^+^ T lymphocytes (33), thereby mitigating such outcomes as lymphopenia. This could also be applied over an extended duration to assist in the treatment of so-called COVID-19 “long haulers” (51). Moreover, the synergistic effect of inhibiting cytokine production and preventing destruction of T lymphocytes by increasing peripheral blood lymphocyte levels could also block overproduction of cytokines capable of suppressing an inflammatory reaction and the life-threatening pro-inflammatory cytokine storm (36). Additionally, as is known for copper and other chlorophyll *a* compounds, SCC may also act as an anti-viral agent (15). Therefore, maintaining homeostasis of the haematopoietic production of peripheral blood leukocytes, primarily of lymphocytes (46), may reduce the likelihood of disease progression to extreme severity.

## Discussion

Drugs to correct lymphopenia do exist but they are not widely available and must be administered under strictly controlled conditions (21). These experimental treatments carry the risk of unwanted side-effects, some of which are severe and even fatal (21). Such therapies can cause destruction of alveolar sacks and thus are unsuitable for the treatment of COVID-19 patients. In contrast, easily manufactured from fescue grass (*Festuca arundinacea*) as a green-black free-flowing powder, SCC is extremely well-tolerated in the diet of adults and children (2, 5, 10). In addition, this profile is unlikely to vary with therapeutic dose, as even at extremely high concentrations no toxicity is reported (8, 10). While indicated to be neither teratogenic nor embryo-lethal in a murine model (52), further research is needed to investigate the dose dependency of any effects (53), and thus to determine if SCC is safe to take when pregnant or breastfeeding. This proviso aside, the utmost consideration should be given to conducting clinical trials to treat COVID-19 patients in the convalescent phase using SCC. This is because it is evident that low peripheral blood leukocyte levels due to primary SARS-CoV-2 infection, and possibly reinfection (54), play a major role in compromising the recovery of individuals with symptomatic COVID-19.

## Conclusion

Therapeutic doses of SCC have been demonstrated to provide an effective clinical treatment for leukopenia. On this basis, we propose that taking SCC at the onset of symptoms or, for immunocompromised patients, at the time of diagnosis, could reverse the lymphopenia observed during COVID-19. It is envisaged that in symptomatic individuals SCC treatment could control leukocyte homeostasis, specifically of lymphocytes, thereby preventing their progressive reduction that is associated with severe disease outcomes. By first restoring and then maintaining adequate peripheral blood levels of CD4^+^ and CD8^+^ T lymphocytes this would enable the immune system of an SCC-treated COVID-19 patient to respond appropriately to resolve SARS-CoV-2 infection. Additionally, it may produce a synergistic effect as SCC is known to block expression of the pro-inflammatory cytokine IL-6. Hence, importantly, such SCC therapy would avoid triggering the characteristically excessive inflammation that causes lasting lung epithelial cell damage and cytokine storm events which often precipitate a fatal outcome of COVID-19.

## Author Contributions

NC and AT-R each made substantial contributions to the conception of the work and to literature search, contributed significantly to writing the manuscript, revised it critically for important intellectual content, approved its final version, and agreed to its submission. Both authors contributed to the article and approved the submitted version.

## Conflict of Interest

The authors declare that the research was conducted in the absence of any commercial or financial relationships that could be construed as a potential conflict of interest.

## Abbreviations

COVID-19: Corona Virus Disease-2019
SARS: Severe Acute Respiratory Syndrome
SARS-CoV-2: Coronavirus (CoV)-2
IL: interleukin
SCC: sodium copper chlorophyllin.

## References

[1] Taiz L Zeiger E. Chapter 7: Photosynthesis: the light reactions. In: Plant Physiology. 5th ed. Sunderland, MA: Sinauer Associates (2010). p. 163–98.

[2] Ferruzzi MG Blakeslee J. Digestion, absorption, and cancer preventative activity of dietary chlorophyll derivatives. Nutr Res. (2007) 27:1–12. 10.1016/j.nutres.2006.12.003

[3] Ulbricht C Bramwell R Catapang M Giese N Isaac R Le TD. An evidence-based systematic review of chlorophyll by the Natural Standard Research Collaboration. J Diet Suppl. (2014) 11:198–239. 10.3109/19390211.2013.859853 24670123

[4] Mysliwa-Kurdziel B Solymosi K. Phycobilins and phycobiliproteins used in food industry and medicine. Mini Rev Med Chem. (2017) 17:1173–93. 10.2174/1389557516666160912180155 27633748

[5] Hayes M Ferruzzi MG. Update on the bioavailability and chemopreventative mechanisms of dietary chlorophyll derivatives. Nutr Res. (2020) 81:19–37. 10.1016/j.nutres.2020.06.010 32828967

[6] Ferruzzi MG Failla ML Schwartz SJ. Sodium copper chlorophyllin: *in vitro* digestive stability and accumulation by Caco-2 human intestinal cells. J Agric Food Chem. (2002) 50:2173–9. 10.1021/jf010869g 11902975

[7] Gomes BB Barros SBM Andrade-Wartha ERS Silva AMO Silva VV Lanfer-Marquez UM. Bioavailability of dietary sodium copper chlorophyllin and its effect on antioxidant defence parameters of Wistar rats. J Sci Food Agric. (2009) 89:2003–10. 10.1002/jsfa.3681

[8] Egner PA Muñoz A Kensler TW. Chemoprevention with chlorophyllin in individuals exposed to dietary aflatoxin. Mutat Res. (2003) 523–4:209–16. 10.1016/S0027-5107(02)00337-8 12628519

[9] Sudakin DL. Dietary aflatoxin exposure and chemoprevention of cancer: a clinical review. J Toxicol Clin Toxicol. (2003) 41:195–204. 10.1081/CLT-120019137 12733859

[10] Gao F Hu XF. Analysis of the therapeutic effect of sodium copper chlorophyllin tablet in treating 60 cases of leukopenia. Chin J Integr Med. (2005) 11:279–82. 10.1007/BF02835789 16417778

[11] Gerić M. Gajski G Mihaljević B Miljanić S Domijan AM Garaj-Vrhovac V. Radioprotective properties of food colorant sodium copper chlorophyllin on human peripheral blood cells *in vitro*. Mutat Res. (2019) 845:403027. 10.1016/j.mrgentox.2019.02.008 31561900

[12] Machhi J Herskovitz J Senan AM Dutta D Nath B Oleynikov MD. The natural history, pathobiology, and clinical manifestations of SARS-CoV-2 infections. J Neuroimmune Pharmacol. (2020) 15:359–86. 10.1007/s11481-020-09944-5 32696264 PMC7373339

[13] Clark NF Taylor-Robinson AW. COVID-19 therapy: could a chlorophyll derivative promote cellular accumulation of Zn^2+^ ions to inhibit SARS-CoV-2 RNA synthesis? Front Plant Sci. (2020) 11:1270. 10.3389/fpls.2020.01270 32922431 PMC7457044

[14] Ito A Tsuneki A Yoshida Y Ryoke K Kaidoh T Kageyama S. *In vitro* inhibition of cytopathic effect of influenza virus and human immunodeficiency virus by bamboo leaf extract solution and sodium copper chlorophyllin. Yonago Acta Med. (2016) 59:61–5. 27046952 PMC4816750

[15] Cortes AA Zuñiga JM. The use of copper to help prevent transmission of SARS-coronavirus and influenza viruses. A general review. Diagn Microbiol Infect Dis. (2020) 98:115176. 10.1016/j.diagmicrobio.2020.115176 33069048 PMC7428768

[16] Wessels I Maywald M Rink L. Zinc as a gatekeeper of immune function. Nutrients. (2017) 9:1286. 10.3390/nu9121286 29186856 PMC5748737

[17] Wu D Lewis ED Pae M Meydani SN. Nutritional modulation of immune function: analysis of evidence, mechanisms, and clinical relevance. Front Immunol. (2019) 9:3160. 10.3389/fimmu.2018.03160 30697214 PMC6340979

[18] Atkinson RL Dahms WT Bray GA Jacob R Sandstead HH. Plasma zinc and copper in obesity and after intestinal bypass. Ann Intern Med. (1978) 89:491–3. 10.7326/0003-4819-89-4-491 697228

[19] Percival SS. Neutropenia caused by copper deficiency: possible mechanisms of action. Nutr Rev. (1995) 53:59–66. 10.1111/j.1753-4887.1995.tb01503.x 7770185

[20] Hantaweepant C Chinthammitr Y Siritanaratkul N. Anemia and neutropenia in copper-deficient patients: a report of two cases and literature review. J Med Assoc Thai. (2016) 99:732–6. 29901325

[21] Bagwe SM Kale PP Bhatt LK Prabhavalkar KS. Herbal approach in the treatment of pancytopenia. J Complement Integr Med. (2017) 14:/j/jcim.2017.14.issue-1/jcim-2016-0053/jcim-2016-0053.xml. 10.1515/jcim-2016-0053 28195548

[22] Poujois A Djebrani-Oussedik N Ory-Magne F Woimant F. Neurological presentations revealing acquired copper deficiency: diagnosis features, aetiologies and evolution in seven patients. Intern Med J. (2018) 48:535–40. 10.1111/imj.13650 29034989

[23] Vyas D Chandra RK. Thymic factor activity, lymphocyte stimulation response and antibody producing cells in copper deficiency. Nutr Res. (1983) 3:343–9. 10.1016/S0271-5317(83)80084-0

[24] Bonham M O'Connor JM Hannigan BM Strain JJ. The immune system as a physiological indicator of marginal copper status? Br J Nutr. (2002) 87:393–403. 10.1079/BJN2002558 12010579

[25] Cakic M Mitic Z Nikolic G Savic I Savic IM. Design and optimization of drugs used to treat copper deficiency. Expert Opin Drug Discov. (2013) 8:1253–63. 10.1517/17460441.2013.825245 23919882

[26] Fang GQ Zhu AP Xu Y. Effect of sodium copper chlorophyllin in treating leukopenia. Anhui Med J. (2001) 22:58–9. 16417778

[27] Hao JP Yan WS. Effect of sodium copper chlorophyllin tablet in treating leukopenia induced by anti-thyroid drugs. J Prac Med. (2004) 20:205.

[28] He XL. Clinical observation of sodium copper chlorophyllin tablet in treating 54 cases of neutropenia in children. J Chin Phys 6: 272.

[29] Huang I Pranata R. Lymphopenia in severe coronavirus disease-2019 (COVID-19): systematic review and meta-analysis. J Intensive Care. (2020) 8:36. 10.1186/s40560-020-00453-4 32483488 PMC7245646

[30] Coronavirus Resource Center, Johns Hopkins University. COVID-19 Dashboard. Available online at: https://coronavirus.jhu.edu/map.html (accessed February 16, 2021).

[31] Terpos E Ntanasis-Stathopoulos I Elalamy I Kastritis E Sergentanis TN Politou M. Hematological findings and complications of COVID-19. Am J Hematol. (2020) 95:834–47. 10.1002/ajh.25829 32282949 PMC7262337

[32] Zhao Q Meng M Kumar R Wu Y Huang J Deng Y. Lymphopenia is associated with severe coronavirus disease 2019 (COVID-19) infections: a systemic review and meta-analysis. Int J Infect Dis. (2020) 96:131–5. 10.1016/j.ijid.2020.04.086 32376308 PMC7196544

[33] Zheng M Gao Y Wang G Song G Liu S Sun D. Functional exhaustion of antiviral lymphocytes in COVID-19 patients. Cell Mol Immunol. (2020) 17:533–5. 10.1038/s41423-020-0402-2 32203188 PMC7091858

[34] Rose-John S. Interleukin-6 signalling in health and disease. F1000Res. (2020) 9:F1000 Faculty Rev-1013. 10.12688/f1000research.26058.1

[35] Mahmud-Al-Rafat A Majumder A Taufiqur Rahman KM Mahedi Hasan AM Didarul Islam KM Taylor-Robinson AW. Decoding the enigma of antiviral crisis: Does one target molecule regulate all? Cytokine. (2019) 115:13–23. 10.1016/j.cyto.2018.12.008 30616034 PMC7129598

[36] Praveen D Puvvada RC Muthukumar VA. Lymphopenia as a marker for disease severity in COVID-19 patients: a metaanalysis. Asian Pac J Trop Med. (2020) 13:426–8. 10.4103/1995-7645.290588

[37] Mahmud-Al-Rafat A Muzammal Haque Asim M Taylor-Robinson AW Majumder A Muktadir A Muktadir H. A combinational approach to restore cytokine balance and to inhibit virus growth may promote patient recovery in severe COVID-19 cases. Cytokine. (2020) 136:155228. 10.1016/j.cyto.2020.155228 32822911 PMC7428755

[38] Tan L Wang Q Zhang D Ding J Huang Q Tang YQ. Lymphopenia predicts disease severity of COVID-19: a descriptive and predictive study. Sig Transduct Target Ther. (2020) 5:33. 10.1038/s41392-020-0159-1 32296069 PMC7100419

[39] Abebe EC Dejenie TA Shiferaw MY Malik T. The newly emerged COVID-19 disease: a systemic review. Virol J. (2020) 17:96. 10.1186/s12985-020-01363-5 32641059 PMC7341708

[40] Fathi N Rezaei N. Lymphopenia in COVID-19: therapeutic opportunities. Cell Biol Int. (2020) 44:1792–7. 10.1002/cbin.11403 32458561 PMC7283672

[41] Hu J Zhou J Dong F Tan J Wang S Li Z. Neutrophil-to-lymphocyte ratio on admission predicts in-hospital mortality in patients with COVID-19. Research Square [Preprint]. (2020). 10.21203/rs.3.rs-49294/v1

[42] Chen Z Wherry JE. T cell responses in patients with COVID-19. Nat Rev Immunol. (2020) 20:529–36. 10.1038/s41577-020-0402-6 32728222 PMC7389156

[43] Qun S Wang Y Chen J Huang X Guo H Lu Z. Neutrophil-to-lymphocyte ratios are closely associated with the severity and course of non-mild COVID-19. Front Immunol. (2020) 11:2160. 10.3389/fimmu.2020.02160 32983180 PMC7493648

[44] Zidar DA Al-Kindi SG Liu Y Krieger NI Perzynski AT Osnard M. Association of lymphopenia with risk of mortality among adults in the US general population. JAMA Netw Open. (2019) 2:e1916526. 10.1001/jamanetworkopen.2019.16526 31790569 PMC6902755

[45] Dhar D Mohanty A. Gut microbiota and Covid-19 – possible link and implications. Virus Res. (2020) 285:198018. 10.1016/j.virusres.2020.198018 32430279 PMC7217790

[46] Sharma D Sandur SK Raghu R Sainis KB. T helper cell differentiation (Th1/Th2) during recovery from radiation-induced lymphopenia and its modulation by chlorophyllin. Bhabha Atomic Research Centre Newsletter. (2009) 309:266–75.

[47] Yin L-M Jiang H-F Wang X Qian X-D Gao R-L Lin X-J. Effects of sodium copper chlorophyllin on mesenchymal stem cell function in aplastic anemia mice. Chin J Integr Med. (2013) 19:360–6. 10.1007/s11655-012-1210-z 23001462

[48] Zheng H You Y Hua M Wu P Liu Y Chen Z. Chlorophyllin modulates gut microbiota and inhibits intestinal inflammation to ameliorate hepatic fibrosis in mice. Front Physiol. (2018) 9:1671. 10.3389/fphys.2018.01671 30564133 PMC6288434

[49] Suryavanshi S Sharma D Checker R Thoh M Gota V Sandur SK. Amelioration of radiation-induced hematopoietic syndrome by an antioxidant chlorophyllin through increased stem cell activity and modulation of hematopoiesis. Free Radic Biol Med. (2015) 85:56–70. 10.1016/j.freeradbiomed.2015.04.007 25872101

[50] Yun CH Jeon YJ Yang Y Ju HR Han SH. Chlorophyllin suppresses interleukin-1 beta expression in lipopolysaccharide-activated RAW 264.7 cells. Int Immunopharmacol. (2006) 6:252–9. 10.1016/j.intimp.2005.08.012 16399630

[51] Lambert and Survivor Corps. COVID-19 “Long Hauler” Symptoms Survey Report. Indiana University School of Medicine. (2020). Available online at: https://www.survivorcorps.com/s/2020-Survivor-Corps-COVID-19-Long-Hauler-Symptoms-Survey-Report-revised-July-254.pdf.

[52] Leite VS Oliveira RJ Kanno TYN Mantovani MS Moreira EG Salles MJS. Chlorophyllin in the intra-uterine development of mice exposed or not to cyclophosphamide. Acta Sci - Health Sci. (2013) 35:201–10. 10.4025/actascihealthsci.v35i2.12470

[53] García-Rodríguez MC Morales-Ramírez P Altamirano-Lozano M. Effects of chlorophyllin on mouse embryonic and fetal development *in vivo*. Teratog Carcinog Mutagen. (2002) 22:461–71. 10.1002/tcm.10042 12395407

[54] Bermejo-Martin JF Almansa R Menéndez R Mendez R Kelvin DJ Torres A. Lymphopenic community acquired pneumonia as signature of severe COVID-19 infection. J Infect. (2020) 80:e23–e24. 10.1016/j.jinf.2020.02.029 32145214 PMC7133663

